# A Security Mechanism for Cluster-Based WSN against Selective Forwarding

**DOI:** 10.3390/s16091537

**Published:** 2016-09-20

**Authors:** Hai Zhou, Yuanming Wu, Li Feng, Daolei Liu

**Affiliations:** School of Optoelectronic Information, University of Electronic Science and Technology, Chengdu 610054, China; 15527238315@163.com (H.Z.); 18328004563@163.com (L.F.); daolei_liu@163.com (D.L.)

**Keywords:** selective-forwarding, inspector node, reputation value, IDS, cluster-based WSN

## Abstract

A wireless sensor network (WSN) faces a number of outsider and insider attacks, and it is difficult to detect and defend against insider attacks. In particular, an insider selective-forwarding attack, in which the attackers select some of the received packets to drop, most threatens a WSN. Compared to a distributed WSN, a cluster-based WSN will suffer more losses, even the whole network’s destruction, if the cluster head is attacked. In this paper, a scheme solving the above issues is proposed with three types of nodes, the Cluster Head (CH), the Inspector Node (IN) and Member Nodes (MNs). The IN monitors the CH’s transmission to protect the cluster against a selective-forwarding attack; the CH forwards packets from MNs and other CHs, and randomly checks the IN to ascertain if it works properly; and the MNs send the gathered data packets to the CH and evaluate the behaviors of the CH and IN based on their own reputation mechanism. The novelty of our scheme is that in order to take both the safety and the lifespan of a network into consideration, the composite reputation value (CRV) including forwarding rate, detecting malicious nodes, and surplus energy of the node is utilized to select CH and IN under the new suggested network arrangement, and the use of a node’s surplus energy can balance the energy consumption of a node, thereby prolonging the network lifespan. Theoretical analysis and simulation results indicate that the proposed scheme can detect the malicious node accurately and efficiently, so the false alarm rate is lowered by 25.7% compared with Watchdog and the network lifespan is prolonged by 54.84% compared with LEACH (Low Energy Adaptive Clustering Hierarchy).

## 1. Introduction

WSN security has drawn much attention over the last few decades. Security plays a vital role in both distributed WSN and cluster-based WSN, especially the cluster-based WSN, because the cluster will be destroyed if the cluster head is attacked by insider attacks [1] or outsider attacks. Compared to outsider attacks, insider attacks are difficult to detect by security mechanisms. In consequence, increasing attention has been drawn to study them. 

Insider attacks cannot be detected and defended against by standard encryption and authentication mechanisms. Insider attacks are launched by “normal nodes” which have been captured by attackers and have acquired a legal identity. Selective forwarding [1] is the hardest identify in insider attacks, in that some core information like reputation value and the threshold of the trust mechanism have been acquired by the malicious nodes and they drop packets in the routing intelligently. For example, they may drop all packets, random packets, regular packets and so on. Due to its flexibility and covertness, it is very difficult to distinguish whether the packet drop is caused by the malicious nodes or the poor condition of the radio channel. Once the selective-forwarding attack launched by the malicious nodes cannot be detected, more vital information will be dropped, which will lead to immeasurable losses or even destroy the entire network.

**Figure 1:**
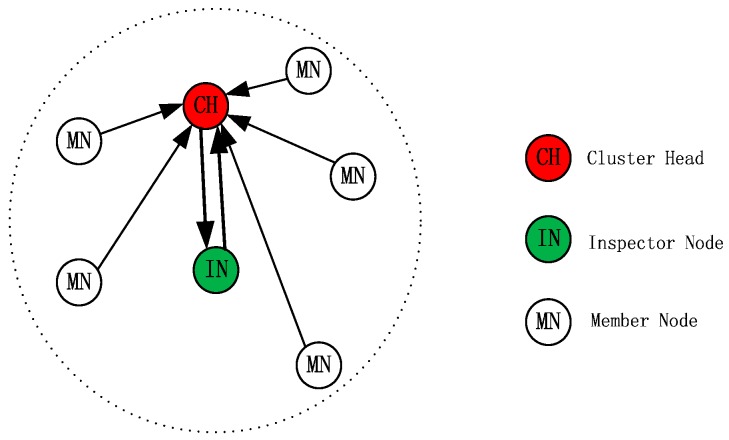
The basic topological structure of a cluster.

In this paper, a new scheme, based on a special node per cluster, an inspector node (IN), is proposed for cluster-based WSNs to provide security against insider selective-forwarding attacks. The proposed scheme consists of three types of nodes working in a specific way in each cluster. Nodes in a cluster are divided into one Cluster Head (CH), one Inspector Node (IN) and Member Nodes (MNs) as shown in Figure 1. The radius of the cluster is half of the communication radius of the nodes so that any two nodes in the same cluster can communicate with each other. The IN monitors the CH’s transmission, and calculates and adjusts the CRV of CH. IN will mark the CH as a malicious node if it finds any malicious behavior during the CH’s transmission and finally informs MNs in the same cluster to remove the CH from their routing table. However, the MNs will not follow the IN’s decision blindly. Except for gathering data, MNs make the right decision about the CH’s behaviors based on what they have overheard and on their own reputation mechanism. Meanwhile, the CH sends random checking packets to the IN during each turn of data collection to make sure the IN is working correctly.

Moreover, in order to balance the node’s energy consumption, thereby prolonging the network lifespan, the node’s surplus energy is introduced on the basis of considering the node’s forwarding rate, which detects malicious nodes to make up the composite reputation value (CRV), which is in turn exploited to determine whether a node will be selected as a CH or IN. Correspondingly, a new method of network arrangement is put forward. Combined with it, the proposed scheme maximally extends the network lifespan.

The remainder of this paper is organized as follows: in Section 2, we review existing works. Section 3 describes the proposed security scheme in detail. Section 4 discusses the method to solve the issue of network lifespan. Section 5 analyzes the simulations and corresponding results. Section 6 concludes this paper. 

## 2. Related Work

Security problems have always been a challenge in the development of WSNs, especially insider attacks [1]. Gulhane and Mahajan [2] described the black hole attack, the most basic insider selective-forwarding attack, dropping all the packets attracted from the forwarding path by itself. This attack can be detected easily, because the malicious node does not forward any packet for a long time, so its neighbor nodes consider it to have failed and decide to update the routing information. An on/off attack [3] is a kind of highly stealthy selective-forwarding attack; its performance is all packet loss or part of packet loss at regular intervals while the rest of the time just acting as normal nodes. Lu et al. [4] point out that selective forwarding is very cunning; malicious nodes, with the purpose of tampering with the content of the packets or dropping parts of interesting packets, select some packets to forward to effectively reduce the possibility of being suspected. Youngho et al. [1] described a collusion attack of multiple nodes, and this kind of attack will inflict massive harm on the whole network.

When analyzing the security issues in WSN, most people will design an intrusion detection system (IDS) [5] based on monitoring and reputation value. The highly representative works are Marti et al. [6] proposing the Watchdog mechanism and Chen-Yang et al. [7] proposing a neighbor-based monitoring mechanism. These mechanisms utilize the direct or indirect trust value between neighbor nodes to judge a node, whether malicious or not, and, currently, many new security mechanisms are designed through improving them.

Liao and Ding [8] suggested a trust mechanism for identifying and removing malicious nodes established between the sending node and its one-hop neighbor nodes, a hybrid continuous strategy monitor-forward game, and it can effectively reduce the error rate of packet loss detection in unreliable radio channels. Hu [9] designs a security mechanism based on monitoring nodes, which are energy-heterogeneous nodes with the function of only monitoring but not forwarding, to suppress the malicious nodes’ insider attacks.

Plenty of new algorithms supported by other theories have also been suggested and studied to solve the security problem; they transfer the focus from identifying the malicious nodes to other effective methods. Biru and Shanchieh [10] focus on the node reliability estimate to restrain the selective-forwarding. They take advantage of the modeling method, by means of a sorting algorithm that can estimate the most suspected nodes and then avoid them in the routing, to analyze the forwarding behaviors of the nodes so that the selective-forwarding can be deeply understood, and finally establish a high quality data-forwarding path. Stavrou and Pitsillides [11] put emphasis on the importance of system recovery after suffering an insider selective-forwarding attack. The mechanism will rebuild a stronger, more reliable and more responsive system with the aid of directional antennas and re-routing based on a blacklist.

## 3. Proposed Security Scheme

We studied the state of the art network security mechanism and found they can effectively improve the network security and, especially, can suppress the insider attacks in WSN well. However, they cannot settle the problem caused by the unbalanced energy consumption of the network very well, such as the “energy hole” problem. As a crucial factor, the energy consumption in a WSN cannot be ignored by any security mechanism because of the finite and non-renewable nature of the energy resource in a WSN. It is a great challenge we must face the issue of how to prolong the lifespan of the network through optimizing the energy consumption balance of the nodes based on the premise of guaranteeing the security of the WSN, and finally set up a safe and sustainable WSN.

**Figure 2:**
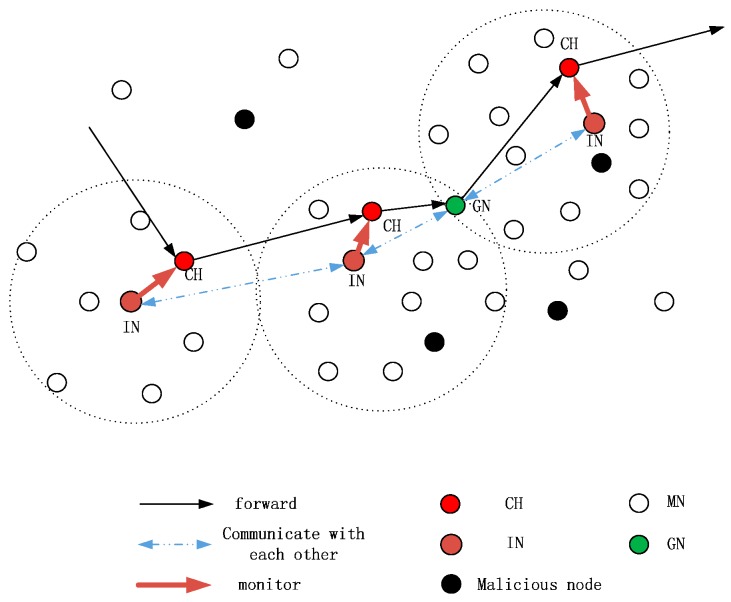
Local monitoring.

Thus, a new security scheme based on IN and CRV is proposed as shown in Figure 2, and we will detail it in this section.

### 3.1. Structure of the Cluster

Different from general cluster, the proposed cluster in this paper consists of three kinds of isomorphic nodes, i.e., Cluster Head (CH), Inspector Node (IN) and Member Node (MN). Meanwhile, the cluster’s radius is equal to half of the nodes’ communication radius to make sure any two nodes in the same cluster can communicate. i.e.,

(1) $$r_{0}=R_{0}/2$$

where *r*_0_ is the radius of the cluster and *R*_0_ is the communication radius of the node. The basic topological structure of the cluster is shown in Figure 1.

### 3.2. Composite Reputation Value (CRV)

The CRV determining function of each node, the maximal one selected as CH and the second one selected as IN, is made up of the node’s forwarding rate and surplus energy as follows:

(2) $$Val\left[node_{id}\right]=a*Pr_{id}+b*\frac{E_{else}}{E_{0}}$$

where *a* and *b* are parameters and 0 < *a* < 1, 0 < *b* < 1, *a + b =* 1. The *E*_0_ is the node’s initial energy and *E_else_* is the surplus energy of the node; *Val[node_id_]* is CRV of the node; *Pr_id_* is forwarding rate of the node.

Reference [9] described how when a node is observed to forward the packet *s* times and drops the packet *f* times, the beta trust model [12] will assign trust value Tr (0 < Tr < 1) to this node using the following equation:

(3) $$T_{r}=\left(s+1\right)/\left(s+f+2\right)$$

We use the beta trust model for *Pr_id_* in our simulation, i.e., *Pr_id_* = Forward_pks / Receive_pks. 

### 3.3. Function of the Node

As shown in Figure 1, the biggest difference is that a special function node IN is added in the cluster. Certainly, the function of CH and MN is also different from in general. The differences are as described below.

#### 3.3.1. Inspector Node (IN)

**Figure 3:**
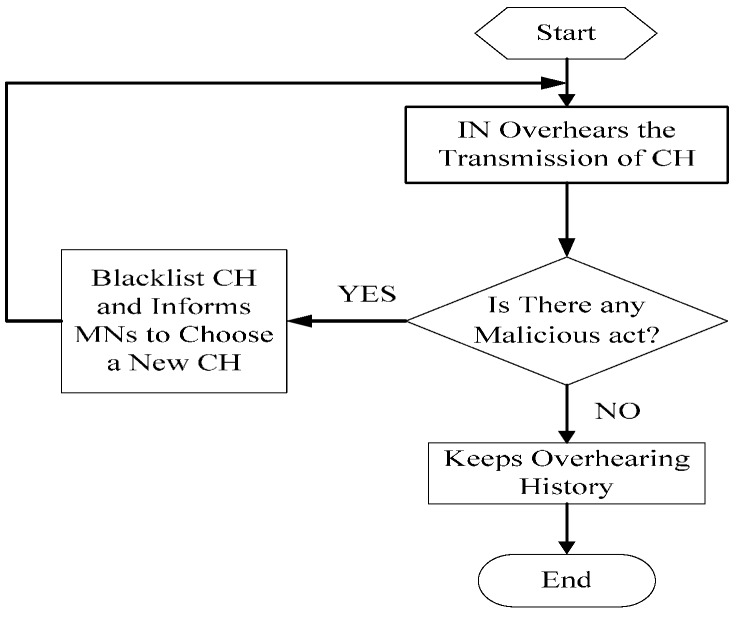
IN’s inspection of the CH.

CH plays an extremely crucial role in cluster-based WSN, not only because it is on behalf of the cluster, but it must act as a Relay Node (RN) when located closer to a sink node. One can imagine the immeasurable losses the CH will bring to the entire network if it is a malicious node. In order to avoid this situation, an IN added in each cluster can serve as a vital defense barrier. Overhearing the transmission of the CH, if IN finds any malicious behavior from CH, IN marks CH as a malicious node directly and informs MNs in the same cluster to remove CH from the routing table of the cluster, and then chooses a new CH from MNs. The workflow of IN is given in Figure 3.

In consideration of IN’s vital function in the proposed scheme, its correct function must be ascertained. Therefore, IN is requested to keep the transmission overhearing history for a fixed duration so that it can satisfy the CH’s random checking. IN removes all previous history after satisfying each random checking request and starts to keep the overhearing history again to satisfy the next random checking request. In this way, IN can be ascertained as working properly.

Absolutely, IN has lots of other important functions inside of the cluster as follows:

1. Broadcasting one packet containing the information (such as node’s ID, hops etc.) of the CH and itself to outside of the cluster for routing between clusters when clustering;
2. Overhearing the MNs’ behaviors to adjust their information during the task of gathering data and evaluating whether they are malicious nodes or not;
3. Communicating with other INs in its routing table to evaluate the CH’s CRV accurately at the end of each period;
4. Broadcasting one packet containing the information of every node in the cluster inside to select a new CH and IN for the next period after the communication between INs.

#### 3.3.2. Cluster Head (CH)

**Figure 4:**
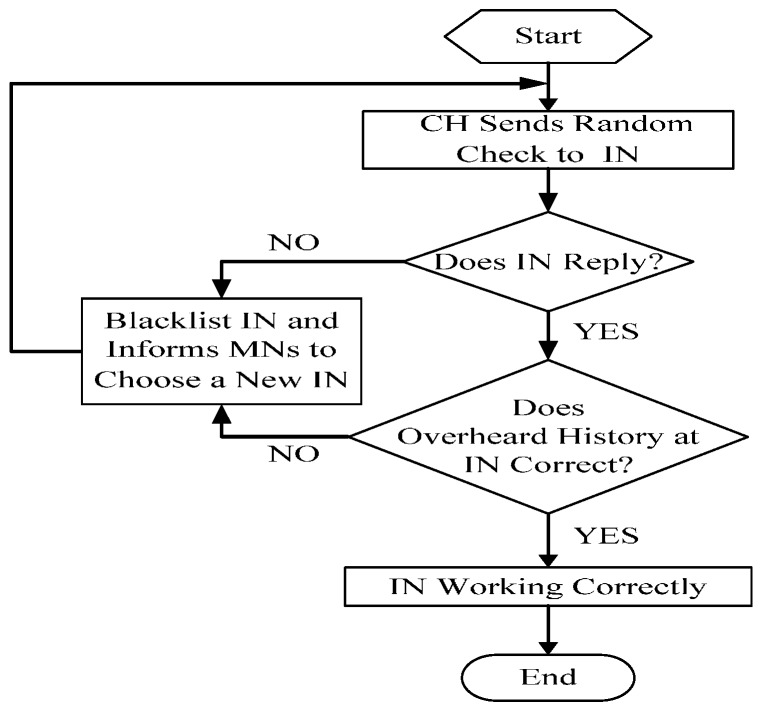
CH’s random check for IN.

As described above, CH does act as a key role in the whole network. Thus, IN is added in each cluster to guarantee the CH’s operating normally. On the contrary, IN should be monitored as well because of the critical task it has to implement. So, in the proposed scheme, CH must check IN by randomly requesting a specific packet the IN has overheard. If the IN has been working correctly, it could answer the request correctly. Since IN cannot know in advance which packet will be requested, it cannot cheat the CH’s random checking process. The workflow is shown in Figure 4.

#### 3.3.3. Member Node (MN)

According to the above description, both CH and IN play a vital role in the network. To MN, it should also evaluate the security of the cluster by itself rather than just gathering data. 

Inside of each cluster, MN evaluates the reputation of the CH and IN. For CH, the reputation is proportional to the rate of its packets being successfully delivered. For IN, the reputation is based on how similar the decisions of the IN on the reputation of others are to its own reputation system. For example, if IN accuses CH, but MNs can judge the CH as a cooperative node based on its own reputation system, the IN will be marked as a malicious node by the MNs. On the contrary, if IN does not accuse CH even though many packets are dropped by CH, MNs will also consider the IN a malicious node.

### 3.4. Monitoring Scheme

**Figure 5:**
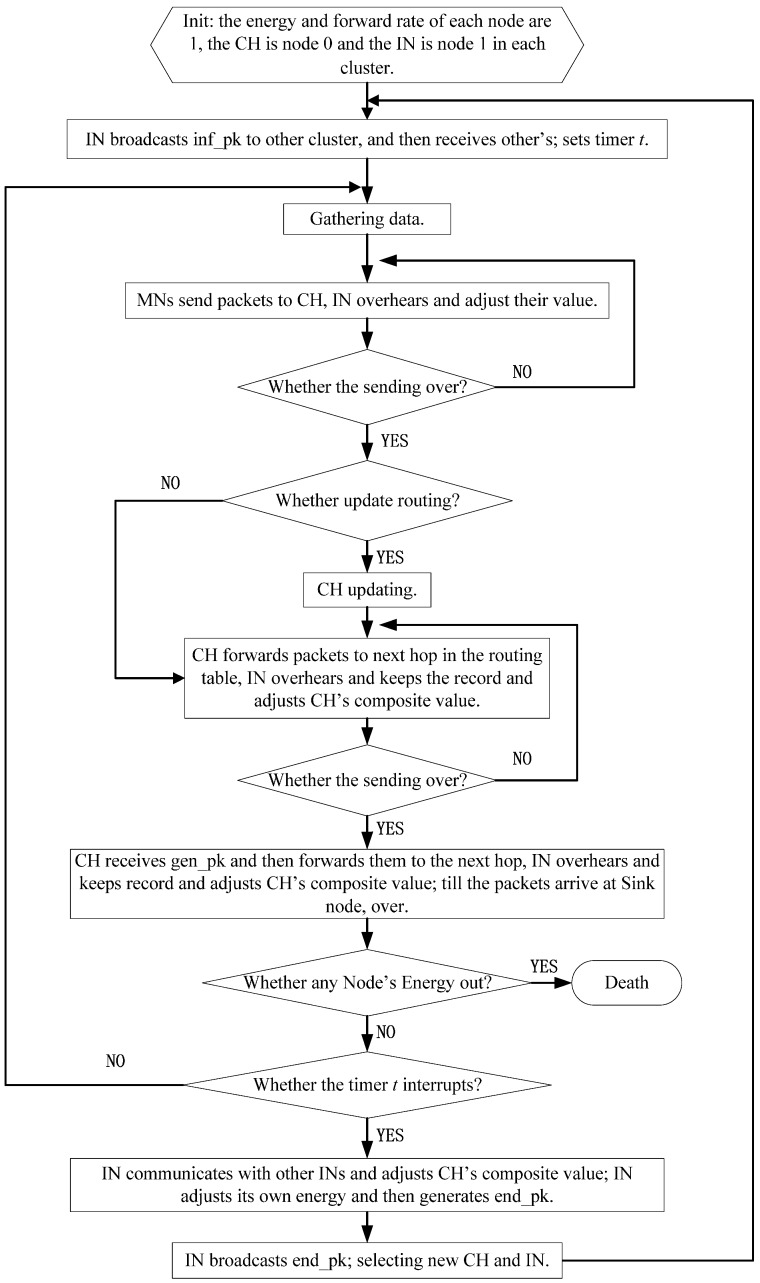
Workflow of local network.

Figure 5 shows the workflow of data forwarding and monitoring in a local network. In the proposed scheme, the stipulations are as follows:

(a) The length of each period is *T* and each period includes several turns of data collection;
(b) The head of data packet should include the related (source, current hop, next hop) ID of CH and IN;
(c) A node overhearing the transmission just needs to extract the head information of the packet.

### 3.5. Functions of the Proposed Scheme

**Figure 6:**
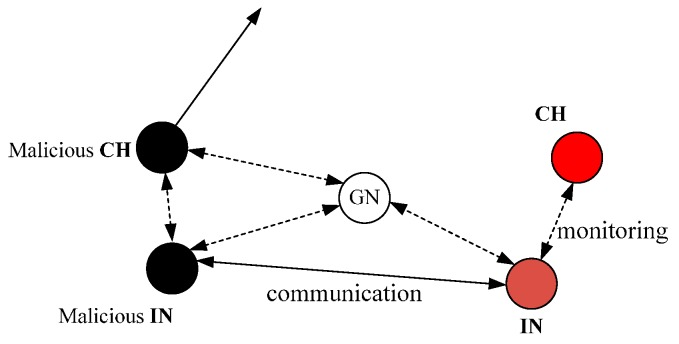
Collusion attack.

The proposed security scheme detailed above can efficiently solve not only a single node’s insider selective-forwarding attack, but multiple nodes’ collusion attacks. Possible conclusions are as follows:

1. Black Hole Attack from One Node. When a node has been captured and become a malicious node, it would launch a black hole attack, i.e., receiving and dropping all the packets. In consequence, its *E_else_* determining the CRV will be high, and then it will be selected as CH, leading to more packets loss. However, the IN can monitor its behavior and will mark it as a malicious node directly.
2. Tampering with Content. When the CH is a malicious node and forwards the received packets after tampering with their content, the IN can overhear it and then marks the CH as a malicious node and informs MNs.
3. Selective Forwarding of One Node. Different from the black hole attack, a node that launches insider selective-forwarding attack cannot be detected easily because it selects parts of packets to drop rather than all of them. In our security scheme, we stipulate that a node will be considered a malicious node if its forwarding rate of one turn is lower than the threshold three times in succession. Therefore, we can detect the malicious node and remove it.
4. Collusion attack. As shown in Figure 6, two malicious nodes collude to cheat other nodes for getting a high CRV so that they can attract more packets and drop all of them. In the proposed scheme, the IN will communicate with other INs in its routing table to accurately evaluate CH’s CRV at the end of each period. At the same time, the communication between the two INs will be overheard by the Gate Node (GN), and then the GN will judge by itself that the CH and IN have colluded to launch an attack in the cluster and informs MNs to remove the CH and IN from their routing table.
5. Versatility. In the proposed security scheme, the CRV is made up of two parts and the different values of *a* and *b* determine different applications of the mechanism. For example, if the monitoring area needs to be observed for a long time, we can make the value of *b* bigger than that of *a* so that the energy consumption will be more balanced and the lifespan of the network will be longer; on the contrary, we can make the value of *a* bigger than that of *b* so that the network will be more reliable.

In this paper, we mainly aim at security issues in a WSN, and, absolutely, the safety of the network should be considered first. However, if we want to set up an efficient WSN with sustainable performance, the energy consumption of nodes must be taken into consideration as a critical factor. As for how to balance the energy consumption and how to ascertain the period *T* to maximally prolong the lifespan of the network, we will discuss that in the next section.

## 4. Network Arrangement

In the proposed scheme, the surplus energy of a node constitutes the vital part of the CRV; it determines the CH’s selection thus determines the network lifespan. Accordingly, in order to solve the problem of an unbalanced node’s energy consumption that limits the network lifespan, a new method of network arrangement is put forward that can make the energy consumption of each node achieve a relatively optimal balance, thereby prolonging the lifespan of the network as much as possible. Next, we will detail that method.

### 4.1. Clustering the Network

**Figure 7:**
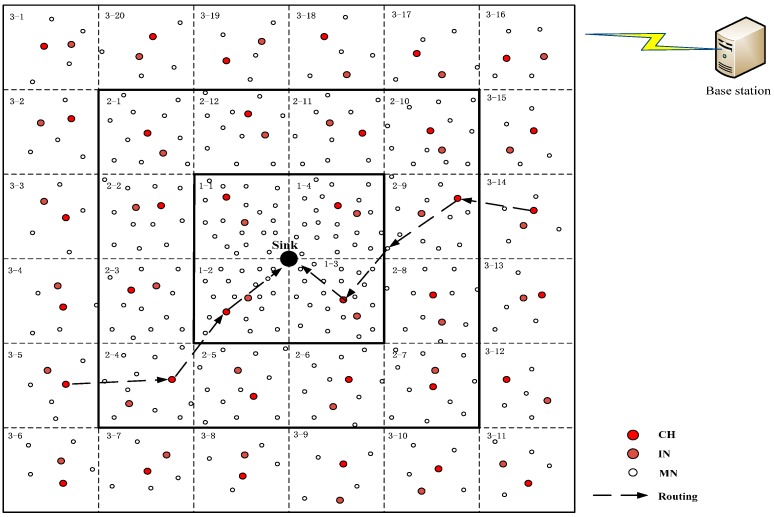
Clustering.

As shown in Figure 7, the monitoring area is divided into several clusters, each of which is a square with the length of its diagonal equal to *R*_0_, based on the geography location information and then each cluster is marked with a number to define the routing direction, and this structure remains constant.

### 4.2. Analysis of Energy Consumption of Node

After clustering, nodes will be distributed in the monitoring area followed the rule that the density of the nodes’ distribution is inversely proportional to their hop, and nodes with the same hop are randomly, uniformly distributed. 

Analysis shows that the smaller the hop of the node, the greater its energy consumption. Because the nodes should not only forward their own packets but also be Relay Nodes (RN) when they are closer to the sink node, their energy will soon run out as a consequence, and followed by the problem of an “energy hole”. Wang [13] exploits energy heterogeneous nodes to solve the problem. Zhang et al. [14] proposes a method of unequal scale clustering based on the location and local density of the nodes to balance the energy consumption of network. Ma et al. [15] studies the model of node distribution and suggests the network arrangement should be based on the hop of the nodes. She combines this with the energy heterogeneous nodes to enhance the pertinence of energy consumption balance of the network.

Combining the above solutions’ advantages and the degree of difficulty in actual operation, a new method of network arrangement is suggested. It is composed of isomorphic nodes and operates easily.

#### 4.2.1. Initialization

When initializing, the initial energy of each node is set to ***E*_0_** = 1 and the energy consumption of receiving and sending one packet is set to ***E_r_*** = 0.0001 and ***E_t_*** = 0.0003, based on the fact that the energy consumption of the node receiving one bit of data compared with that of sending one bit data is 1:2.7 [9]. Compared to the CPU, the energy consumption of receiving and sending data takes a large proportion in the whole energy consumption, so we stipulate the surplus energy of the node is determined by communication energy consumption in the proposed scheme.

According to the analysis detailed in the Section 4.2, the reason causing the “energy hole” problem should be taken into consideration when carrying out the network layout. What this means is that more nodes should be distributed to share the additional tasks of acting as RN in a smaller hop area rather than evenly distributed in the whole monitoring area. Therefore, in the proposed method of network arrangement, more nodes are added in the smaller hop area to balance the extra relay task. As shown in Figure 7, the network is divided into three layers based on nodes’ hop: the first layer is distributed by one-hop nodes and includes four clusters with the biggest density of node distribution, and the average number of nodes is ***m*_1_** per cluster; the second layer is distributed by two-hop nodes and includes twelve clusters, its density of nodes distribution is second and the average number of nodes is ***m*_2_** per cluster; the third layer is distributed by three-hop nodes and includes twenty clusters with the smallest density of nodes distribution, and the average number of nodes is ***m*_3_** per cluster. ***m*_1_** > ***m*****_2_** > ***m*****_3_**; the routing direction of the data is from the third layer to the second layer and then to the first layer and finally to the sink node.

#### 4.2.2. Proportion of Node Distribution Density

In Figure 7, the area of each cluster is equal, so the required number of nodes just gathering data during one turn in each cluster is also equal. This means we use the same number of nodes (the number is ***m*_3_**) to gather data while the other MNs will be in a sleep state during one turn in each cluster.

For the third layer, each cluster just needs to accomplish its own task of gathering data during one turn, so the analysis of energy consumption (the CH and IN don’t need to sense data) is as follows:

(1) Each MN sends *5* packets to the CH during one turn, the sum of the energy consumption is
  $$\left(m_{3}-2\right)\times 5E_{t}=5\left(m_{3}-2\right)E_{t};$$
(2) The CH receives all the packets, then fuses and compresses them to 30% and sends out, the sum of the energy consumption is
  $$\left(m_{3}-2\right)\times 5E_{r}+\left(m_{3}-2\right)\times 5\times 30\%\times E_{t}\;=1.5\left(m_{3}-2\right)E_{t}+5\left(m_{3}-2\right)E_{r}\;;$$
(3) The IN overhears all the process, the sum of the energy consumption is
  $$\left(m_{3}-2\right)\times 5E_{r}+\left(m_{3}-2\right)\times 5\times 30\%\times E_{r}=6.5\left(m_{3}-2\right)E_{r};$$

Therefore, the total energy consumption of each cluster in the third layer during one turn is

(4) $$E_{3}=6.5\left(m_{3}-2\right)E_{t}+11.5\left(m_{3}-2\right)E_{r}$$

For the second layer, each cluster needs to accomplish the additional task except for its own task (the energy consumption is ***E*_3_**). According to Figure 7, we can calculate that each cluster in the second layer has to undertake an average of *(20*/*12)* clusters’ task from the third layer during one turn. Thus the analysis of energy consumption is as follows:

(1) The CH acts as RN to forward packets from other clusters, the sum of the energy consumption is
  $$\left(\frac{20}{12}\right)\times 1.5\left(m_{3}-2\right)\times \left(E_{r}+E_{t}\right)=2.5\left(m_{3}-2\right)\left(E_{r}+E_{t}\right);$$
(2) The IN overhears the process that the CH acts as RN, the sum of the energy consumption is
  $$\left(\frac{20}{12}\right)\times 1.5\left(m_{3}-2\right)\times E_{r}=2.5\left(m_{3}-2\right)E_{r}\;$$

Therefore, the total energy consumption of each cluster in the second layer during one turn is

(5) $$E_{2}=2.5\left(m_{3}-2\right)\left(E_{r}+E_{t}\right)+2.5\left(m_{3}-2\right)E_{r}+E_{3}=9\left(m_{3}-2\right)E_{t}+16.5\left(m_{3}-2\right)E_{r}$$

For the first layer, each cluster needs to accomplish the additional task except for its own task (the energy consumption is ***E*_3_**). According to Figure 7, we can calculate that each cluster in the first layer has to undertake an average of (12/4) clusters’ task from the second layer during one turn. Thus the analysis of energy consumption is as follows:

(1) The CH acts as RN to forward packets from other clusters, the sum of the energy consumption is
  $$\left(\frac{12}{4}\right)\times \left[\left(\frac{20}{12}\right)\times 1.5\left(m_{3}-2\right)\times \left(E_{r}+E_{t}\right)+1.5\left(m_{3}-2\right)\times \left(E_{r}+E_{t}\right)\right]=12\left(m_{3}-2\right)\left(E_{r}+E_{t}\right);$$
(2) The IN overhears the process that the CH acts as RN, the sum of the energy consumption is
  $$\left(\frac{12}{4}\right)\times \left[\left(\frac{20}{12}\right)\times 1.5\left(m_{3}-2\right)\times E_{r}+1.5\left(m_{3}-2\right)\times E_{r}\right]=12\left(m_{3}-2\right)E_{r};$$

Therefore, the total energy consumption of each cluster in the second layer during one turn is

(6) $$E_{1}=12\left(m_{3}-2\right)\left(E_{r}+E_{t}\right)+12\left(m_{3}-2\right)E_{r}+\;E_{3}=18.5\left(m_{3}-2\right)E_{t}\;+35.5\left(m_{3}-2\right)E_{r}$$

As is known, the lifespan of the network is determined by the first dead node. For the entire network, only in the ideal situation that every node in the network is dead due to running out of energy at the same time will the lifespan of the network be longest. So, if the network lifespan is to be prolonged to the utmost, the energy consumption of every node in the network should be the same. This is Equation (7),

(7) $$\frac{E_{3}}{m_{3}}=\frac{E_{2}}{m_{2}}=\frac{E_{1}}{m_{1}}$$

Combining Equations (4)–(7) result in

(8) $$m_{3}\;:m_{2}\;:\;m_{1}=\;1\;:1.41\;:2.94$$

Absolutely, ***m*_1_**, ***m*_2_**, and ***m*_3_** are integers. We set ***m*_3_** *=* *6* (too large will produce unnecessary waste, too small cannot cover the whole cluster; 1 CH, 1 IN, 4 MN), then ***m*_2_** *=* *9*, and ***m*_1_** *=* *18*.

Consequently, the proportion of the node distribution density from the third layer to the first layer is 1:1.5:3.

### 4.3. Analysis of the Lifespan of the Network

According to the analysis above, the total energy consumption of the entire network during one turn is

(9) $$E_{oneturn}=4E_{1}+12E_{2}+20E_{3}=1248E_{t}+2280E_{r}$$

The total energy of the entire network is

(10) $$E=\left(18\times 4+9\times 12+6\times 20\right)E_{0}=300E_{0}$$

Generally, the length of the network lifespan has a direct causal relationship with the total energy of the entire network as well as the energy of a single node. Next, both the total energy consumption of each layer and the energy consumption of a single node will be analyzed to make it clear how to prolong the lifespan of the network maximally.

#### 4.3.1. Energy Consumption of Changing CH and IN

In the proposed security scheme, the energy consumption of changing CH and IN in each cluster is made up of three aspects:

(a) IN communicates with other INs in its routing table. It sends a packet to its previous hop IN (no need if there is none) in its routing table, and receives a packet from its next hop IN (no need if there is none) in its routing table later, assuming the energy consumption is ***E_a_***.
(b) IN broadcasts a packet containing the information of all the nodes in the cluster to the inside of the cluster. After receiving, the nodes update the corresponding information stored before. The energy consumption is assumed to be ***E_b_***.
(c) IN broadcasts a packet outside of the cluster after changing the CH and IN. Later, IN receives packets from other clusters whose hop is smaller, assuming the energy consumption is ***E_c_***.

Then, we analyze the average energy consumption of single cluster in each layer.

The third layer, because the hop of this layer is the biggest, so the clusters in this layer just need to receive packets from the second layer and need not send packets outside. Thus, the energy consumption of a single cluster in this layer about the above three aspects is

(11) $$\{\begin{array}{c} E_{a3}=E_{r}\;\;\;\;\;\;\;\;\;\;\;\;\;\;\;\;\;\;\\ \;E_{b3}=E_{t}+5E_{r}\;\;\;\;\;\;\;\;\\ E_{c3}=\frac{44}{20}E_{r}\;\;\;\;\;\;\;\;\;\;\;\;\;\;\; \end{array}$$

On the second layer, each cluster in this layer needs to send packets to outside and receive packets from the first layer. Thus, the energy consumption of single cluster in this layer about the above three aspects is

(12) $$\{\begin{array}{c} E_{a2}=E_{t}+E_{r}\;\;\;\;\;\;\\ E_{b2}=E_{t}+8E_{r}\;\;\;\;\\ \;\;E_{c2}=E_{t}+\frac{20}{12}E_{r}\;\;\;\; \end{array}$$

In the first layer, because the hop of this layer is the smallest, so the clusters in this layer just need send packets outside and need not receive packets from other clusters. Thus, the energy consumption of a single cluster in this layer for the above three aspects is

(13) $$\{\begin{array}{c} E_{a1}=E_{t}\;\;\;\;\;\;\;\;\;\;\;\;\;\;\;\;\\ E_{b1}=E_{t}+17E_{r\;}\;\\ E_{c1}=E_{t}\;\;\;\;\;\;\;\;\;\;\;\;\;\;\;\;\; \end{array}$$

Consequently, the total energy consumption of the entire network to change the CHs and INs once is shown in Equation (14),

(14) $$E_{refresh}=4\times \left(E_{a1}+E_{b1}+E_{c1}\right)+12\times \left(E_{a2}+E_{b2}+E_{c2}\right)+20\times \left(E_{a3}+E_{b3}+E_{c3}\right)=68E_{t}+360E_{r}$$

Assuming that one period, from the network starting to the accomplishment of changing the CHs and INs once, includes *n* turns, i.e., changing the CHs and INs once every *n* turns, the whole network can change the CHs and INs *N* times. Then, the total energy consumption of the entire network during one period is

(15) $$E_{T}=n\times E_{oneturn}+E_{refresh}$$

and the relationship between *E* and *E_T_* is shown in Equation (16),

(16) $$N\times E_{T}\leq E$$

Now, so that the energy consumption of the node is more balanced and thereby the lifespan of the network becomes longer, the value of *n* and *N* must be, at the same time, their maximum and integer, which can make the lifespan of the network its longest if all the nodes in the network are dead due to running out of energy at the same time after *N* periods, *n* turns per one period, of work. However, in order to get the optimal value of the *n* and *N*, it is not enough only to analyze the total energy consumption of the entire network. Therefore, the energy consumption of a single node will be analyzed in the next part.

#### 4.3.2. Energy Consumption of a Single Node

According to the above data, in the third layer, the average energy consumption of gathering data in one turn of single node during one period is $\left[E_{3}+\left(E_{a3}+E_{b3}+E_{c3}\right)/n\right]/6$, then getting the Equation (17),

(17) $$n\times N\times \left[E_{3}+\left(E_{a3}+E_{b3}+E_{c3}\right)/n\right]/6\leq E_{0}$$

In the second layer, the average energy consumption of gathering data in one turn of single node during one period is $E_{2}+\left(E_{a2}+E_{b2}+E_{c2}\right)/n]/9,\;$then getting the Equation (18),

(18) $$n\times N\times \left[E_{2}+\left(E_{a2}+E_{b2}+E_{c2}\right)/n\right]/9\leq E_{0}$$

In the first layer, the average energy consumption of gathering data in one turn of single node during one period is $E_{1}+\left(E_{a1}+E_{b1}+E_{c1}\right)/n]/18$ then getting the Equation (19),

(19) $$n\times N\times \left[E_{1}+\left(E_{a1}+E_{b1}+E_{c1}\right)/n\right]/18\leq E_{0}$$

### 4.4. Conclusion

Summarizing the Section 4.2 and Section 4.3, simultaneous equations show that *n* *=* *9* and *N* *=* *54*. In this case, the energy consumption of a single node in each cluster is the most balanced and the lifespan of the network is the longest.

Therefore, the conclusion of the new method of network arrangement in this paper is that the proportion of the node distribution density from the third layer to the first layer is 1:1.5:3 and the entire network changes the CHs and INs every *9* turns of data collection.

## 5. Simulation Analysis

In this section, we use OPNET 14.5 to conduct a simulation of evaluating the performance of the proposed security scheme, mainly aiming at the detection of the insider selective-forwarding attack and the lifespan of the network. In our simulation, 300 isomorphic nodes are randomly distributed over a 300 m × 300 m area.

### 5.1. Function of Detecting the Malicious Node

For the proposed scheme, we conduct a simulation of local network communication and malicious node detection. Before that, some related conditions need to be set as follows.

Each node’s initial energy is ***E*_0_** *= 1* and the energy consumption of receiving and sending one packet is ***E_r_*** *= 0.0001* and ***E_t_*** *= 0.0003*. The local network consists of two neighboring cluster located in Figure 7, cluster 1 from the second layer contains 9 nodes with the ID from 0 to 8, and cluster 2 from the third layer contains 6 nodes with the ID from 9 to 15. The cluster 1 not only needs to accomplish its own task but also needs to forward the packets from cluster 2. 

We assume 25 s each turn. The first 10 s, each cluster updates its own routing table (go to the next step if needed) and then the CH receives the packets from the MNs and forwards them to the next hop at a speed of 2 packets/s after fusing and compressing, the IN overhears the transmission; the next 13 s, the CH in cluster 1 receives the packets from the CH in cluster 2 and then forwards them, the IN overhears the transmission; the last 2 s, the IN calculates the CRV of the nodes and broadcasts packets.

The forwarding rate of each node is inequality from 70% to 100%. In cluster 1, we assume the forwarding rate of node 0 and 4 is 70%–90%; node 1 and 7 is 80%–100%; node 2 and 5 is 75%–85%; node 3 and 8 is 70%–90%; the initial forwarding rate of node 6 is 70%–100%, after a certain number of turns, it will become a malicious node to launch a selective-forwarding attack with a forwarding rate of 30%–50%.

**Figure 8:**
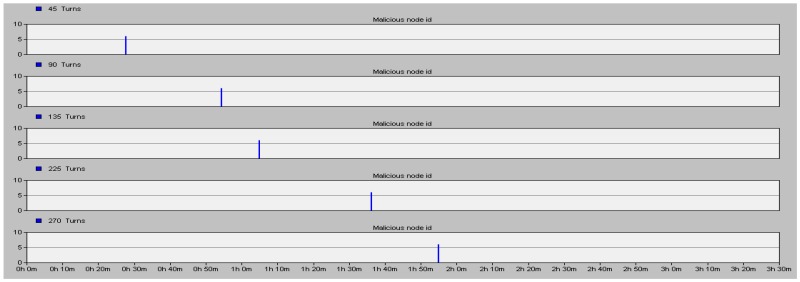
Detection of node 6 captured in different time.

We do a 12,500 s (500 turns) simulation. Figure 8 shows that node 6 becomes a malicious node after being captured in 1125 s, 2250 s, 3375 s, 5625 s and 6750 s and the corresponding time when the IN detects the ID number (y axis) of the malicious node.

The proposed security scheme can detect the malicious node accurately whenever the normal node was captured to be a malicious node as shown in Figure 8. On the other hand, the false alarm rate (the ratio of false alarm times and simulation times) of the proposed scheme is also much better than that of the Watchdog mechanism and the Neighbor-based monitoring mechanism. Results are shown in Table 1.

By comparing, the false alarm rate of the proposed security scheme is lower by 25.7% than that of the Watchdog.

### 5.2. Analysis of Packets Loss Rate

**Figure 9:**
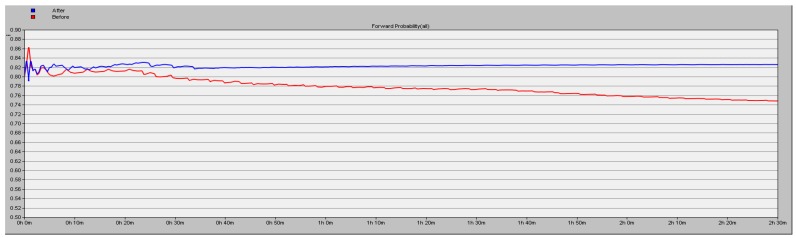
Forward probability of the network with one malicious node.

**Figure 10:**
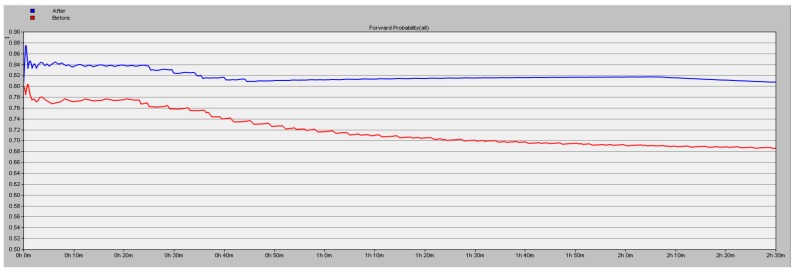
Forward probability of the network with two malicious nodes.

**Figure 11:**
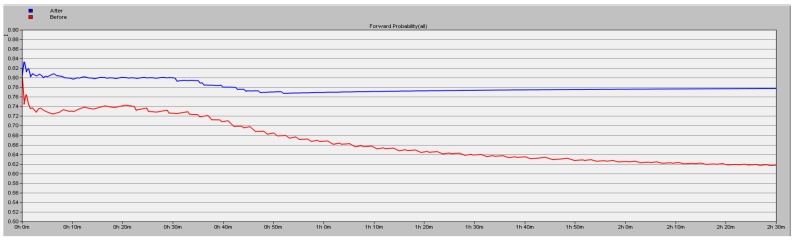
Forward probability of the network with three malicious nodes.

The simulation results detailed in Section 5.1 show the high efficiency of the proposed scheme. In this part, we test about the forward probability (y axis) under the conditions of different number of malicious nodes in the network. We simulate 9000 s and the results are shown in Figure 9, Figure 10 and Figure 11, the blue line corresponding to using the proposed security scheme and the red line corresponding to not using it. A conclusion can be drawn that the packet loss rate is lower by 16.06% in the case of three malicious nodes in the network.

### 5.3. Simulation of Energy Consumption Balance and Lifespan of the Network

In Section 4, a new method of network arrangement corresponding to the proposed security scheme is suggested. Moreover, the feasibility of it to implement the energy consumption balance of the nodes and the lifespan prolongation of the network is proved correct, and the related conclusions are also calculated.

**Figure 12:**
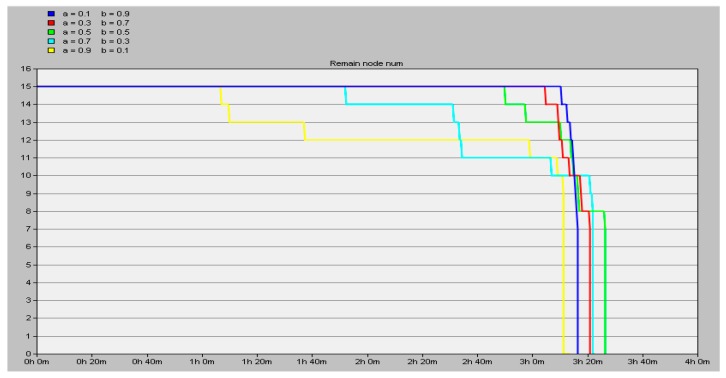
The number of remaining nodes in the network.

The simulation is carried out in two neighboring clusters, a total of 15 nodes, chosen from the second and third layer, one each. When running, the CHs and INs are changed once every 9 turns as a period. According to adjustments of the value of the coefficients *a* and *b* in Equation (2), we simulate for 14,000 s until all the nodes in the network are dead and we get the number of remaining nodes (y axis) in the network as shown in Figure 12.

Observing the data in Figure 12, we conclude that the energy consumption balance of the network is relatively better when the surplus energy of the node serves as a vital factor in choosing the CH and IN, i.e., the value of *b* is greater than that of *a*. Especially when setting *a* = 0.1, *b* = 0.9, all the nodes are nearly dead at the same time (the blue line in Figure 12). In this situation, the energy consumption balance of the nodes achieves the relative optimum and the lifespan of the network is also the longest.

Compared to LEACH regarding the lifespan of the network, as Table 2 shows, the suggested method of network arrangement in this paper is much better. It can be calculated that the network lifespan is prolonged by 54.84%.

## 6. Conclusions

In this paper, a new security scheme based on an inspector node (IN) and composite reputation value (CRV) is proposed for a cluster-based WSN, and it consists of three types of nodes: the Cluster Head (CH), the Inspector Node (IN) and Member Nodes (MNs). Moreover, the CRV including the node’s forwarding rate and surplus energy is exploited to determine the selection of the CH and IN. This is different from a general approach just using the node’s forwarding rate to detect malicious nodes, and combining it with the node’s surplus energy can prolong the network lifespan. Correspondingly, a new method of network arrangement is put forward aiming at balancing the nodes’ energy consumption to prolong the lifespan of the network. Simulation results indicate that the proposed scheme can detect the malicious node accurately, the false alarm rate is lowered by 25.7% compared with Watchdog, and the network lifespan is prolonged by 54.84% compared with LEACH.

## Figures and Tables

**Table 1:** False alarm comparison.

Mechanism	Type of Threshold	Threshold	Number of Malicious Node	Simulation Times	Detection Times	False Alarm Times	False Alarm Rate
Watchdog	Static	0.7	1	1000	961	282	28.2%
Neighbor-based monitoring	Static	0.7	1	1000	975	172	17.2%
Proposed	Dynamic	Init 0.7	1	1000	998	25	2.5%

**Table 2:** Network lifespan comparison.

Mechanism	Simulation Environment	Simulation Times	Network Lifespan (Average Turns)
LEACH	Local network	1000	480.405
Proposed	Local network	1000	310.259

## References

[1] Cho Y.H., Qu G., Wu Y.M. Insider Threats against Trust Mechanism with Watchdog and Defending Approaches in Wireless Sensor Networks. Proceedings of the 2012 IEEE Symposium on Security and Privacy Workshops (SPW).

[2] Gulhane G., Mahajan N. (2014). Performance Evaluation of Wireless Sensor Network under Black Hole Attack. Int. J. Comput. Technol..

[3] Chae Y., Dipippo L.C. (2015). Trust Management for Defending On-Off Attacks. IEEE Trans. Parallel Distrib. Syst..

[4] Lu Z., Sagduyu Y., Li J. Queuing the Trust: Secure Backpressure Algorithm against Insider Threats in Wireless Networks. Proceedings of the 2015 IEEE Conference on Computer Communications (INFOCOM).

[5] Butun I., Morgera S.D., Sankar R. (2014). A Survey of Intrusion Detection Systems in Wireless Sensor Networks. IEEE Commun. Surv. Tutor..

[6] Marti S., Giuli T.J., Lai K., Baker M. Mitigating Routing Misbehavior in Mobile and Ad Hoc Networks. Proceedings of the International Conference on Mobile Computing and Networking (Mobicom).

[7] Tseng C.Y., Balasubramanyam P., Ko C., Limprasittiporn R., Rowe J., Levitt K. A Specification-Based Intrusion Detection System for AODV. Proceedings of the the 1st ACM Workshop on Security of Ad Hoc and Sensor Networks.

[8] Liao H., Ding S. (2015). Mixed and Continuous Strategy Monitor-Forward Game Based Selective Forwarding Solution in WSN. Int. J. Distrib. Sens. Netw..

[9] Hu Y., Wu Y.M., Wang H.S. (2014). Detection of Insider Selective Forwarding Attack Based on Monitor Node and Trust Mechanism in WSN. Wirel. Sens. Netw..

[10] Cui B., Yang S.J. NRE: Suppress Selective Forwarding Attacks in Wireless Sensor Networks. Proceedings of the 2014 IEEE Conference on Communications and Network Security (CNS).

[11] Stavrou E., Pitsillides A. Recovering from The Attack in WSNs Enhancing The Recovery Benefits of Blacklisting and Rerouting Using Directional Antennas. Proceedings of the 2014 International Wireless Communications and Mobile Computing Conference (IWCMC).

[12] Josang A., Ismail R. The Beta Reputation System. Proceedings of the 15th Bled Electronic Commerce Conference.

[13] Wang H.S., Wu Y.M., Hu Y. (2015). An Energy-balanced Routing Algorithm on Heterogeneous Deployment in WSN. J. Inf. Comput. Sci..

[14] Zhang Q., Chai Q. (2011). Unequal Scaled Clustering Routing for WSN Based on Redundancy of Cluster Headers. Comput. Eng..

[15] Ma L., Tong L., Ma D.C. (2015). A Kind of Node Distribution Strategy for the Energy Hole Problem of WSNs. Comput. Meas. Control.

